# mHealth Technologies for the Care of Children With Congenital Heart Disease: Scoping Review

**DOI:** 10.2196/83385

**Published:** 2026-07-17

**Authors:** Mingzhu Wang, Qiuyin Pan, Shengrong Tan, Yuanchun Kong, Zhengling Dai, Jiao Cai, Duoqin Bi, Jiali Zhou

**Affiliations:** 1 Children's Heart Center, Guizhou Branch of Shanghai Children’s Medical Center, Shanghai Jiao Tong University School of Medicine, Guizhou Hospital, Guiyang, Guizhou, China, 86 13601626175; 2 Shanghai Children's Medical Center Affiliated to Shanghai Jiaotong University School of Medicine, Shanghai, China

**Keywords:** congenital heart disease, mobile health, scoping review, pediatric, telemedicine

## Abstract

**Background:**

Mobile information technology (IT) is increasingly being used in the health care sector, and it can play a critical role in both the care of children with congenital heart disease (CHD) and the quality of life of their families.

**Objective:**

This study aimed to conduct a scoping review of the application of mobile health (mHealth) technologies in the care of children with CHD. We summarized the forms of mHealth interventions and effects on CHD to provide a reference for future research in this field.

**Methods:**

We searched PubMed; Embase; Web of Science; the Cochrane Library; CINAHL; China National Knowledge Infrastructure; Wanfang Data; the Chinese Biomedical Database; VIP Chinese Science and Technology Journal Database; National Guideline Clearinghouse of the United States; the website of the Registered Nurses’ Association of Ontario, Canada; the Guidelines International Network; the American Heart Association; and the American Association of Cardiovascular and Pulmonary Rehabilitation. The search period was from the establishment of the databases to June 12, 2025. The retrieved literature was screened and analyzed.

**Results:**

A total of 519 Chinese- and English-language articles were identified, with 44 (8.5%) studies meeting the inclusion criteria. The primary forms of mHealth interventions for patients with CHD included mobile apps, wearable devices, and remote monitoring equipment. The findings indicated that mHealth technologies could improve exercise capacity, nutritional status, psychological well-being, and quality of life in children with CHD.

**Conclusions:**

The application of mHealth in the care of children with CHD is feasible and demonstrates positive effects. Future research should emphasize peer education and patient privacy protection while further exploring remote education and health management based on theoretical frameworks and intelligent ITs to enhance quality of life for both children with CHD and their parents.

## Introduction

Congenital heart disease (CHD) is a heterogeneous group of structural abnormalities in the cardiovascular system with an incidence rate of 7–9 per 1,000, ranking as the most common birth defect. Worldwide, over 1 million infants are born with CHD each year, and China reports 150,000 to 200,000 new cases annually [1-3]. CHD comprises a complex and diverse spectrum of conditions with severe consequences, imposing substantial economic burdens and psychological distress on families and society. As a major public health concern, CHD significantly impacts children’s physical and mental health, as well as their quality of life.

Traditional face-to-face intervention models are often constrained by medical costs, travel distances, and time limitations, making it difficult to provide timely and effective personalized, precise, and continuous health care services. These constraints negatively impact patient health management. In contrast, mobile health (mHealth) apps transcend temporal and spatial barriers, ensuring both the continuity and accessibility of medical resources while motivating patients to actively engage in their own health management. Guidelines indicate that remote comonitoring of cardiac patients’ cardiopulmonary rehabilitation by both families and health care professionals is feasible, safe, and effective [4]. The use of mHealth technology for home monitoring of children with CHD can help identify clinical issues, prevent unnecessary emergency visits, ensure child safety, and positively impact caregivers’ psychological well-being and health care use [5,6]. Although research on the application of mHealth technology in children with CHD has been conducted both domestically and internationally, it remains in the early stages. This study aimed to summarize the applications and effects of mHealth technology in the care of children with CHD, providing a reference to promote its further development in this field.

To address this gap in the literature, this study used a scoping review methodology to examine the application of mHealth technology in children with CHD. The specific objectives were to (1) systematically identify the application of forms of mobile IT in the nursing care of children with CHD; (2) summarize the effects of mobile IT on the exercise function, nutritional status, psychological state, and quality of life of these children; and (3) identify gaps in the existing research. This study aimed to highlight the limitations of the current literature and provide directions for future research on mHealth-supported self-management for children with CHD.

## Methods

### Literature Search Strategy

This study used a scoping review methodological framework to systematically map the current research landscape on mHealth management for children with CHD, identifying key concepts, evidence types, and knowledge gaps. Given the limited number of studies on the specific application of mHealth technologies in this pediatric population, this review aimed to delineate the overall research landscape rather than evaluate the effectiveness of specific interventions; therefore, a scoping review was chosen over a systematic review. This report adheres to the PRISMA-ScR (Preferred Reporting Items for Systematic Reviews and Meta-Analyses extension for Scoping Reviews) checklist. We systematically searched multiple databases from their inception to June 12, 2025, including international databases (PubMed, Embase, Web of Science, the Cochrane Library, the website of the Registered Nurse and CINAHL), Chinese databases (China National Knowledge Infrastructure, Wanfang Data, the Chinese Biomedical Database, and VIP Chinese Science and Technology Journal Database), and guideline repositories (Guidelines International Network, National Guideline Clearinghouse, National Institute for Health and Care Excellence, Scottish Intercollegiate Guidelines Network, the Canadian Medical Association clinical practice guideline, World Health Organization, American Heart Association, American Association of Cardiovascular and Pulmonary Rehabilitation, and Medlive). The search strategy for this study was structured around 3 core conceptual modules: the target population (children with CHD), the intervention measures (mHealth and digital technologies), and the research context (nutrition, exercise, management, monitoring, and education). In the target population module, we combined terms for “congenital heart disease.” For the intervention measure module, keywords including “mobile health,” “mHealth,” “telemedicine,” “smartphone,” “mobile application,” and “WeChat” were used. The modules were combined using the Boolean operator “AND.”

This search strategy was first developed for the PubMed database and subsequently adapted for other databases according to their controlled vocabularies (eg, MeSH [Medical Subject Headings] terms) and syntax rules. Although the search strings varied across different databases, the core conceptual structure remained consistent to ensure the comprehensiveness and reproducibility of the search. The search strategy can be found in Multimedia Appendix 1.

### Literature Inclusion and Exclusion Criteria

Inclusion criteria were (1) studies focusing on mHealth technologies for children with CHD, (2) a study population consisting of patients with CHD aged 18 years or younger, and (3) publications in Chinese or English. Exclusion criteria were (1) duplicate publications; (2) unavailable full-text articles; and (3) case reports, conference abstracts, or similar publication types.

### Study Selection and Data Extraction Process

Literature screening and data extraction were independently conducted by 2 reviewers. First, the 2 reviewers independently performed title and abstract screening and full-text screening based on the inclusion and exclusion criteria; any disagreements were resolved through discussion or by consulting a third reviewer. Subsequently, the 2 reviewers independently extracted data using a predesigned structured data extraction form (Microsoft Excel format) and cross-checked the results. The extracted information included first author, publication year, country, study type, specific form of mHealth intervention (eg, apps, remote monitoring platforms, and social media), intervention content (eg, nutritional management, exercise rehabilitation, and parental psychological support), and main study findings. During the extraction process, any discrepancies were resolved through discussion to reach a consensus.

## Results

### Literature Search Results

**Figure 1.**
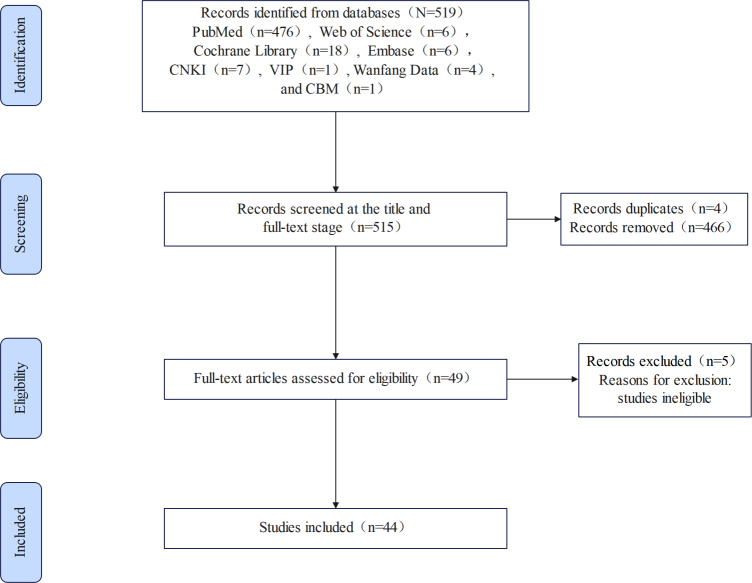
Flowchart of the article screening process. CBM: Chinese Biomedical Database; CNKI: China National Knowledge Infrastructure.

A preliminary search yielded a total of 519 relevant articles (n=476, 91.7% from PubMed; n=6, 1.2% from Web of Science; n=18, 3.5% from the Cochrane Library; n=6, 1.2% from Embase; n=7, 1.3% from China National Knowledge Infrastructure; n=1, 0.2% from VIP Chinese Science and Technology Journal Database; n=4, 0.8% from Wanfang Data; and n=1, 0.2% from the Chinese Biomedical Database). All articles were imported into a reference management software for deduplication. Of the 519 retrieved articles, after removing 4 (0.8%) duplicates, 515 (99.2%) remained for title and abstract screening. Following the review of titles and abstracts, of the 515 articles, 466 (90.5%) that were clearly irrelevant were excluded, leaving 49 (9.5%) for full-text review. During the full-text screening phase, of the 49 articles, 5 (10.2%) that did not meet the inclusion criteria were excluded, resulting in a final inclusion of 44 (89.8%) articles, as shown in Figure 1 and Multimedia Appendix 2 [7-50].

### Basic Characteristics of the Included Studies

#### Study Types and Country Distribution

Of the 44 included studies, 24 (54.5%) were intervention studies, including 9 (20.5%) randomized controlled trials and 6 (13.6%) observational studies. Geographically, 45.5% (n=20) of the studies were from China, mostly focusing on nursing interventions and health education using the WeChat platform; 20.5% (n=9) of the studies were from the United States; and 6.8% (n=3) of the studies were from Norway, emphasizing qualitative and survey research on parental acceptance of mobile apps and psychological support. The remaining studies were from India, the Netherlands, Italy, Japan, the United Kingdom, Germany, and Argentina.

#### Forms of Mobile IT Application

The forms of mobile IT used in the included studies comprised mobile apps for functions such as health education, symptom monitoring, and decision support; wearable devices for the collection of vital signs or physical activity data; and telemedicine platforms—including WeChat, video consultations, and telephone follow-ups—for postdischarge follow-up, home monitoring, or online guidance.

#### Overview of Intervention Content and Effects

The literature systematically demonstrated the broad application trend of mHealth technology in the management of children with CHD. In terms of intervention content, the studies focused on exercise intervention or cardiopulmonary rehabilitation, nutritional management, psychological support, and quality of life. Exercise interventions or cardiopulmonary rehabilitation facilitated the improvement of children’s motor function through remote guidance or monitoring. Nutritional management encompassed breastfeeding guidance, removal of nasogastric tubes, and nutritional risk assessment. Psychological support primarily involved remote education and cognitive behavioral interventions to alleviate anxiety and depression in both children and their parents.

## Discussion

### Principal Findings

This study used a scoping review methodology to systematically examine the current status and effects of mobile IT in the nursing care of children with CHD. The findings indicate that mHealth technologies, primarily in the form of apps, wearable devices, and remote monitoring, demonstrated positive potential in improving exercise function, nutritional status, psychological state, and quality of life in children with CHD. Additionally, these technologies significantly contributed to enhancing parental caregiving capacity and alleviating psychological burden. However, existing research still faces challenges such as high heterogeneity in interventions, lack of theoretical frameworks, privacy and security risks, and unequal accessibility of devices. These issues suggest that this field remains in the early stages of development, and future research requires further standardization and improvement.

### Overview and Current Status of mHealth

mHealth uses mobile phones, apps, and monitoring devices to facilitate communication between patients and pediatric health care professionals, track health-related data, and enable access to pediatric health information. It incorporates functions such as tracking, feedback, and reminders, supporting disease screening, daily activity monitoring, medication adherence, and health education [51]. Unlike traditional telephone-based follow-up methods, mHealth has evolved toward greater specialization, systematization, and intelligence tailored to patients’ disease characteristics and needs [52]. mHealth technology adherence refers to the extent to which health data transmitted by parents to health care providers align with recommended home-based symptom monitoring protocols. Parents with higher adherence to mHealth tools can more promptly identify symptoms in their children, leading to improved health outcomes [53]. Additionally, the mHealth management model offers a flexible and practical approach for remote postdischarge follow-up, diagnosis, and treatment in cardiac patients [54]. Currently, mHealth has become an indispensable medical tool; however, research on its application in CHD care remains limited. This review synthesized evidence on the use of mHealth in CHD management, aiming to provide insights for optimizing and expanding its adoption in pediatric CHD care.

### Application Modalities of mHealth in Children With CHD

#### Mobile Apps

Currently, applications based on mobile phones, computers, and web platforms are widely used for vital sign monitoring, rehabilitation training, follow-up management, and health education in children with CHD. A study found that the implementation of an app integrating functionalities from prenatal diagnosis and neonatal screening to postoperative follow-up reduced the number of deaths among children with CHD from 598 to 353, representing a 41% decrease [7]. A survey also showed that, among 119 parents of children with CHD, 104 (87.4%) were willing to accept follow-up management based on WeChat [8]. Smartphone apps support remote monitoring of vital sign data, including oxygen saturation, blood pressure, temperature, and weight, and can assess medication adherence as well as the incidence and severity of symptoms. Relevant data can be uploaded to hospital information systems, facilitating timely awareness of the child’s condition by medical staff. Furthermore, some apps incorporate integrated decision support systems to assist in formulating or adjusting treatment and nursing plans and provide health education materials in video and PDF formats along with online consultation features [9-11]. For instance, cardiac observation apps, through thoughtful design of content and functionality, effectively supplement traditional medical follow-up systems, helping parents gain more guidance and psychological support when facing their children’s illness [12]. Carlson et al [13] developed a web-based automated follow-up system that automatically sends health status questionnaires to families at 30 days and 1 year after the child’s discharge, aiding in the identification of high-risk patients requiring enhanced follow-up. The study also found a strong demand among families for knowledge related to heart disease and behavioral guidance. Future research could further explore the feasibility of conducting personalized remote rehabilitation, follow-up, and health education based on the severity of the child’s condition and individual needs.

#### Wearable Devices

Current evidence indicates that wearable devices (eg, heart rate monitors, pedometers, and sensors) are primarily worn on the wrist, waist, or chest. These devices can continuously record vital sign parameters such as electrocardiography, heart rate, blood pressure, and oxygen saturation; some devices can also monitor weight changes [10,14]. However, existing studies are characterized by small sample sizes and short follow-up periods, making it difficult to assess long-term effects. Furthermore, the device models, monitored parameters, and intervention protocols vary considerably across studies, hindering cross-study comparability of results. Currently, wearables are predominantly used for monitoring, and there is a lack of integrated management models that combine them with early warning systems and remote interventions. Additionally, issues concerning the accuracy of device data, privacy and security, and children’s compliance with wearing the devices require further investigation. Future research should focus on threshold setting for wearable device parameters and data storage functions. This would enable families to understand their children’s health status in real time while also providing stored information to medical professionals. Such data would offer a basis for understanding children’s condition at home and developing personalized intervention plans.

#### Remote Examination Devices

The application of remote examination devices in the diagnosis and treatment of children with CHD primarily encompasses modalities such as remote auscultation, remote consultation, remote diagnosis, and remote echocardiography. In the realm of remote auscultation, computer-aided auscultation systems, through heart sound acquisition, signal processing, and automated analysis algorithms, can effectively differentiate pathological murmurs, thereby reducing unnecessary referral burden [15]. Digital stethoscopes have also proven effective in identifying clinical issues, with no instances of missed diagnoses reported [16]. A remote diagnosis system in Japan has been successfully implemented for the prenatal diagnosis and pregnancy management of CHD in hospitals located in remote areas [17]. Regarding remote imaging diagnosis, tele-echocardiography has demonstrated accuracy, safety, and efficacy in screening children with CHD, potentially sparing families unnecessary interhospital transfer costs [18]. In terms of teleconsultation, video-based teleconsultation saves time and medical costs for parents of children with CHD [19]. It also allows for modifications to the patient’s treatment plan and facilitates communication regarding subsequent treatment strategies [20].

Despite the significant advantages demonstrated by remote examination devices, several challenges persist. Artificial intelligence–assisted auscultation requires expensive digital stethoscope equipment, and its widespread adoption and application may face economic and technological barriers. The operation of remote examination devices necessitates specialized training; the standardization of procedures and diagnostic consistency among primary health care providers require further evaluation. Furthermore, the lack of unified interoperability and data sharing standards among different devices limits the integrated application of telehealth systems. Future research should focus on reducing device costs, enhancing user-friendliness, and promoting the deployment of remote examination and diagnostic devices in primary care hospitals, thereby extending access to quality medical resources for patients in remote areas.

### Potential of mHealth in Cardiac Rehabilitation

#### Physical Exercise

There is current evidence suggesting that mHealth technologies hold promise for enhancing the accessibility of cardiac exercise therapy and reducing implementation barriers [14]. However, findings regarding intervention effects remain inconsistent. Meyer et al [21] found that a web-based exercise intervention, while demonstrating the safety of 60 minutes of weekly physical activity, did not yield significant improvements in health-related physical fitness or quality of life among children with complex CHD. Conversely, other studies have indicated that wearable device–based cardiopulmonary rehabilitation can improve muscle endurance in children with heart disease [22]. Regarding study design, existing research on exercise interventions exhibits considerable heterogeneity. Variations in intervention duration, exercise modalities, and monitoring devices across studies render direct comparisons and synthesis of results difficult. Furthermore, most studies have small sample sizes and lack long-term follow-up data, precluding assessment of the long-term effects and safety of the interventions. The application of mHealth technologies in the field of exercise rehabilitation for children with CHD is still in an exploratory phase. Although existing evidence preliminarily confirms its safety and feasibility, its effectiveness remains unclear. Future research should conduct large-sample, multicenter randomized controlled trials that incorporate objective measures of physical capacity (eg, cardiopulmonary exercise testing) rather than relying solely on self-reports to validate the effectiveness of different exercise intervention protocols. Personalized remote exercise prescriptions could be developed tailored to children at different developmental stages—such as infants, school-aged children, and adolescents. Establishing a hospital-home remote supervision mechanism ensuring exercise safety under the joint monitoring of family members and medical staff is crucial. Additionally, exploring the integration of exercise monitoring data with early warning systems to enable real-time feedback and dynamic adjustments represents a key direction for future investigation.

#### Nutrition

Mobile ITs have played a positive role in nutritional risk identification and assessment, feeding guidance and nutritional improvement, and liberation from nasogastric tube feeding with associated home management for children with CHD. A telehealth home monitoring program facilitated the early identification of infants with CHD at risk of weight-for-age abnormalities, enabling timely customization of interventions by health care providers for this vulnerable population [23]. In another study, a nutritional risk assessment tool integrated into a mobile app automatically calculated and analyzed children’s nutritional status upon data entry, providing decision support for clinicians to devise personalized nutritional interventions [24]. Multiple studies have demonstrated that telehealth education for parents of infants after CHD surgery—covering disease knowledge, postoperative care, home feeding, and complication management—can significantly enhance parental caregiving competence and improve the infants’ postoperative nutritional status [25,26]. Notably, health education based on the WeChat platform has proven effective in improving breastfeeding efficiency and parental satisfaction for infants after CHD surgery [27]. Furthermore, remote nutritional management has been shown to promote neonatal growth and development while reducing the incidence of respiratory infections and hospital readmission rates [26]. Telehealth interventions grounded in multidisciplinary team collaboration combined with individualized patient assessment and effective communication with parents have facilitated the successful transition from nasogastric tube to oral feeding in children with CHD at home [28]. A WeChat-based discharge follow-up system also improved treatment adherence, enhanced cardiac function and nutritional status, and reduced the incidence of adverse events in these children [29].

Although existing studies generally support the positive effects of mHealth technologies on the nutritional management of children with CHD, several limitations remain. Some studies rely on parent-reported data, which may introduce recall bias, and the heterogeneity of nutritional outcome measures (eg, weight, length and height, BMI, feeding efficiency, and readmission rates) makes it difficult to conduct meta-analyses for integration. The nutritional assessment functions of mHealth tools are mostly limited to data calculation and currently lack deep integration with electronic health record systems or clinical decision support. Children with CHD are at higher nutritional risk than their healthy peers. Future research should establish a core outcome set, evaluate the differential effects of interventions across patients with varying nutritional risk stratification, focus on long-term nutritional follow-up in postoperative patients, explore deep integration of mHealth technologies with clinical nutritional guidelines, and enhance technology accessibility for low-income families.

#### Psychological Outcomes

Mobile ITs have also demonstrated utility in alleviating anxiety and depression and improving the psychological well-being of children with CHD, their parents, and adolescent patients. For pediatric patients, these technologies have a positive impact on reducing anxiety and depression levels [30], with studies focusing on adolescents further confirming that mobile phone–based digital health interventions can enhance emotional well-being in this population [31]. Furthermore, Internet Plus–based health education, while enhancing disease-related knowledge among family members, can indirectly alleviate negative emotions in children and improve cardiac function while reducing complication rates [32]. At the parental level, multiple studies have shown that preoperative and postoperative health education and nursing guidance provided to parents of children with CHD via WeChat effectively ameliorates parental anxiety [33,34]. Nurse-guided mHealth interventions have also been associated with reduced anxiety and depression in parents following a prenatal diagnosis of critical CHD [35]. Additionally, telehealth services such as remote consultations and follow-up care enable timely assessment of the children’s physical condition, reduce the need for hospital visits, and provide professional guidance for home care, thereby alleviating parental stress, anxiety, and depression and reducing caregiving burden [36-38].

However, existing research predominantly focuses on parental psychological states, with relatively fewer studies examining psychological outcomes in pediatric patients themselves, particularly young children. While interventions are diverse—including WeChat-based education, app-based interventions, and nurse-guided support—there is a lack of comparative studies evaluating the efficacy of different intervention modalities or assessing the long-term maintenance of psychological benefits. Furthermore, most studies use generic scales for psychological assessment, lacking condition-specific instruments validated for the pediatric CHD population. Children with CHD face unique psychosocial challenges during the transition to adulthood. Future research should prioritize the psychological health of patients during this critical transitional period, develop mHealth-based support programs for transition care, and explore integrated care models that combine psychological support for children with concurrent parental psychological interventions.

#### Quality of Life

There is current evidence suggesting that mHealth technology shows promising potential in improving quality of life for children with CHD and their parents, particularly demonstrating significant effects in enhancing treatment adherence, reducing complication rates, and alleviating parental caregiving burden. Studies have shown that telemedicine solutions have a positive impact on cardiovascular health [39]. Regarding the quality of life of pediatric patients, postdischarge follow-up management based on mHealth technology has demonstrated favorable intervention outcomes. Research indicates that using a WeChat-based follow-up nursing system can effectively improve out-of-hospital treatment adherence among children, reduce the incidence of adverse cardiac events, and enhance their quality of life [40]. An IT-supported transitional care model helped strengthen parental caregiving capabilities, improve nursing satisfaction, and reduce postoperative complications and readmission rates, thereby improving patient prognosis [41-43]. Remote health education targeting parents has also been associated with reduced complication rates and lower postdischarge loss to follow-up [33,44]. In terms of symptom monitoring, parents’ use of mobile devices to upload daily symptom data facilitates timely identification of abnormalities by health care providers and enables prompt intervention, contributing to more positive health outcomes for children [53].

At the parental level, mHealth technologies have demonstrated multidimensional intervention value. Personalized decision support apps can enhance parents’ understanding of common infant health conditions, assist them in completing health assessments and reports, and improve access to health care services [45]. Health education and psychological counseling delivered via the WeChat platform have proven effective in improving parental caregiving capacity, alleviating anxiety and grief, and reducing caregiving burden, thereby enhancing overall quality of life [46,47]. Individualized cardiac observation apps facilitate communication between parents and health care providers and improve readiness for hospital discharge [48]. Furthermore, WeChat-based guidance for preparing children for transthoracic echocardiography and for postoperative follow-up has been shown to alleviate parental anxiety and increase satisfaction with medication management [38,49]. Research focusing on mothers of children in intensive care units has similarly found that mHealth apps help reduce separation anxiety and improve the extent to which family needs are met [50].

Despite the generally positive findings, some studies have reported inconsistent effects of mHealth interventions. One telehealth home monitoring program, for instance, failed to significantly reduce psychological distress in parents of children with CHD [23]. These differences may be related to the complexity of the children’s CHD and the technical usability of the mobile apps. Interventions targeting parental psychological support require further optimization. Current research exhibits considerable heterogeneity in intervention content, follow-up duration, and outcome measures, and no consensus has been reached regarding the effectiveness of interventions for parental stress. Therefore, current studies lack evidence on long-term quality of life improvements for both pediatric patients and their parents. Moreover, there is selection bias in the study designs—families willing to participate in mHealth research may inherently have higher health literacy and socioeconomic status, leading to overestimated effects [55,56]. Future research should incorporate intention-to-treat analyses and report dropout rates along with reasons for attrition. High-quality studies are still needed to identify optimal intervention strategies and the populations for which they are most appropriate.

### Implementation Barriers: Health System–Level Challenges

Although the studies included in this review reported clinical effects of mHealth technologies, almost none discussed their implementation feasibility in real-world health systems. Below, we analyze barriers from 5 perspectives: cost, workflow integration, data security, equity, and recommendations for translation into practice.

#### Cost and Resource Allocation

Sustainable application of mHealth technologies requires ongoing hardware updates, software maintenance, and human resources. To date, no formal cost-effectiveness analysis of mHealth interventions for children with CHD has been conducted [57]. Moreover, the personnel costs associated with data monitoring are often concealed by research funding. Future studies should explicitly report direct and indirect costs as well as the incremental cost-effectiveness ratio.

#### Integration With Hospital Workflows

If an intervention cannot be integrated into existing workflows, its clinical efficacy loses practical relevance. Existing mHealth programs required nursing staff to periodically review data from apps but did not assess the additional workload. Furthermore, most apps were incompatible with hospital electronic medical record systems, necessitating manual data transcription and increasing the risk of errors and administrative burden [58]. Future research should prioritize platforms that seamlessly interface with electronic medical record systems and adopt automated alert algorithms to reduce manual review.

#### Data Security and Regulatory Compliance

mHealth technologies involve the transmission and storage of pediatric health information, and risks of privacy breaches during data collection, transmission, and storage are common. However, existing studies have inadequately described data encryption, anonymization, and user authorization mechanisms [59]. Future research should explicitly report data security measures in the “Methods” section and recommend developing open-source, locally compliant platforms.

#### Equity and Accessibility

The widespread use of mHealth technologies may exacerbate health inequities, with device costs and digital literacy being 2 core barriers for low-income families [60]. The initial investment in basic monitoring equipment (eg, smartphones and wearable sensors) and data plans may worsen the financial burden for some rural families, especially in the absence of insurance coverage or subsidies. Even when devices are available, insufficient digital literacy among caregivers (eg, inability to install apps or upload data) can compromise intervention effectiveness and adherence. Future research should report household income and baseline digital literacy and explore low-technology alternatives (eg, SMS text messages plus voice follow-up), device rental or subsidy programs, and community-based digital coaching as integrated support models.

#### Recommendations for Translation Into Practice

Future research, while reporting clinical outcomes, should focus on family acceptability, long-term intervention feasibility, nurse workload, and data security. Technologically, it is necessary to improve the accuracy of wearable devices; develop low-cost, high-precision solutions; and strengthen privacy protection and ethical compliance. Attention should be paid to digital accessibility for patients of low socioeconomic status and those in remote areas to narrow the digital divide. Research should be based on theoretical frameworks, include child-reported psychological outcomes and disease-specific measurement tools, and extend follow-up periods. Gamified interventions and qualitative research should be explored to gain an in-depth understanding of user needs.

### Summary

mHealth technology holds positive potential in the nursing care of children with CHD, improving multiple health outcomes and reducing parental burden. Its mechanisms of action may be related to self-efficacy, adherence, and behavior change. Future research should conduct mechanism studies based on behavioral theory to provide a theoretical foundation for optimizing interventions.

## Supplementary material

10.2196/83385 Multimedia Appendix 1 Search strategy used in the PubMed database.

10.2196/83385 Multimedia Appendix 2 Summary of included studies on digital health interventions for congenital heart disease.

10.2196/83385 Checklist 1 PRISMA-ScR checklist.

## Abbreviations

CHD: congenital heart disease
MeSH: Medical Subject Headings
mHealth: mobile health
PRISMA-ScR: Preferred Reporting Items for Systematic Reviews and Meta-Analyses extension for Scoping Reviews
